# Driving sustainable ruminant livestock transformation through the Global Farm Platform: the hub-and-spoke network

**DOI:** 10.1093/af/vfag014

**Published:** 2026-04-07

**Authors:** M Jordana Rivero, Joshua Onyango, GFP Members, Andrew Cartmill, Andrew Cooke, Anne Farrugia, Chinyere Ekine, Claire Morgan-Davies, Daniel Enriquez Hidalgo, Dave Chadwick, Graeme Martin, Hai-Chun Jing, Kalimalwayo Mwakasungula, Katrien Geudens, E M Muhammed, Nicola Lambe, Nicolás López-Villalobos, P T Suraj, Walter Ayala, Yingjun Zhang, Michael R F Lee

**Affiliations:** Farming Footprints and Adaptations, Rothamsted Research, North Wyke, Devon, UK; Promar International, Nantwich, Cheshire, UK; Harper and Keele Veterinary School, Newport, Shropshire, UK; School of Sustainable Food and Farming Harper, Adams University, Newport, Shropshire, UK

**Keywords:** bidirectional innovation, climate resilience, knowledge exchange, One Health, smallholder empowerment

Implications

- The hub-and-spoke network, proven effective in agricultural extension and knowledge dissemination worldwide, empowers the Global Farm Platform (GFP) to translate cutting-edge research into practical, context-specific innovations that enhance ruminant livestock sustainability across diverse global systems.
- By connecting research hubs to commercial spokes, this approach bridges the science-practice gap, delivering economic gains, environmental sustainability, and positive social impact while addressing local challenges like climate resilience and biodiversity loss.
- GFP is scalable through partnerships and networks which informs policy and empowers farmers and smallholders to produce nutritious food with lower impacts, aligning with FAO’s sustainable livestock transformation vision.
- In a resource-constrained world, hub-and-spoke networks offer a resilient blueprint for transformative agriculture, fostering collaboration over isolation for planetary and human health.

## The Hub-and-Spoke Network: Overview and Applications in Agriculture

The hub-and-spoke network model, originating from transportation (e.g., airline systems), features a central hub for coordination and peripheral spokes for localized execution. It promotes efficiency in resource allocation, knowledge sharing, and scaling by reducing redundancies and enabling adaptability, as seen in supply chains.

In agriculture, this structure supports sustainable production, logistics, and technology transfer: central hubs (e.g., research institutions or facilities) develop innovations, which spokes (farmer networks or regional outlets) adapt and apply locally. This is valuable in diverse agroecological settings for enhancing resilience to climate change and optimizing resources like water and nutrients. Nevertheless, highly centralized networks can lead to homogenized practices and limit innovation (Matous and Bodin, 2024); hence, strategically designed hub-and-spoke systems linking research hubs to diverse farmer “spokes” remain indispensable for promoting varied experimentation, resource optimization, and long-term innovation in sustainable agriculture.

A key impact of the hub-and-spoke network in agriculture is its role in translating science into real-world activities. By bridging the gap between researchers and practitioners, it accelerates the adoption of evidence-based technologies, enhances knowledge exchange, and measures effectiveness through metrics like income gains or environmental improvements. Studies highlight how it empowers smallholders via accessible information, reduces barriers to entry for new farmers, and informs policy for broader scalability. However, it must be emphasized that knowledge flow must be two-way, where application, assessment and development of interventions on farm must equally flow to the hub to optimize future understanding and utilize practical knowledge of local practitioners.

## The GFP Hub-and-Spoke Network and Its Relevance to Sustainable Ruminant Systems

**Figure 1.**
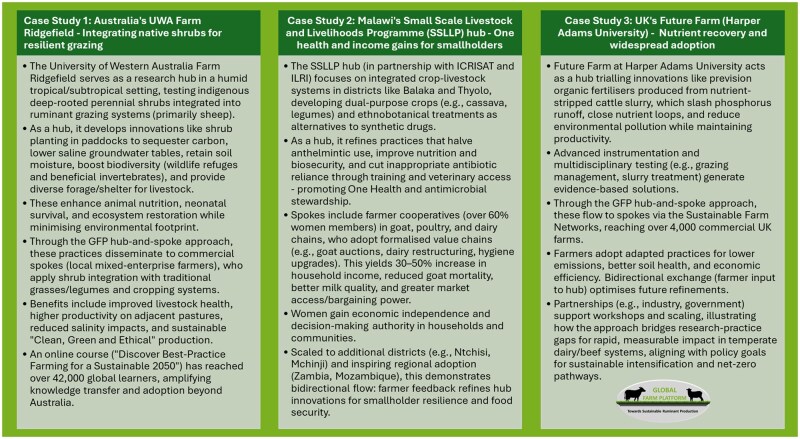
Exemplary case studies from the Global Farm Platform hub-and-spoke approach.

In a world where ruminant livestock systems face mounting pressures from climate volatility, biodiversity loss, animal health and welfare, economic barriers and the demand for nutritious food, innovative approaches are essential. The GFP (globalfarmplatform.org), launched in 2014 (Eisler et al., 2014) and recently honored by the FAO for excellence in sustainable livestock transformation, embodies this through its pioneering hub-and-spoke network (Figure 1). Here, research farms act as “hubs,” living laboratories where scientists test and refine practices, while connected commercial farms or smallholders serve as “spokes,” adapting and disseminating these innovations to real-world contexts. Unlike conventional research stations, which often operate in isolation, the GFP’s hubs function as dynamic living laboratories that collaborate across the global network, integrate advanced instrumentation with practical farmer knowledge, delivering solutions that are context-specific, economically viable and environmentally sustainable. From tropical smallholder systems in Malawi to temperate dairy operations in the UK, the GFP tackles ruminant farming’s core dilemmas: how to feed more people with less impact. In our view, this approach flips the script on traditional top-down research, fostering bidirectional knowledge flow that empowers farmers as co-innovators rather than passive recipients.

## Operating within Socio-ecological Contexts: Addressing Key Challenges

No hub operates in a vacuum; each is embedded in its socio-ecological fabric, turning regional challenges into opportunities for broader sustainability. Take Uruguay’s Palo a Pique hub (INIA): by employing no-tillage rotations, intercropping of pastures and crops, and forage legumes, it enhances soil structure and fertility through biological nitrogen fixation, reducing the intensity of greenhouse gases emissions per hectare while boosting grain yields by up to 23% (Pereyra-Goday et al., 2026). In Australia’s UWA (University of Western Australia) Farm Ridgefield, integration of indigenous shrubs within grazing demonstrate that ruminant grazing can coexist with, and support, ecosystem restoration, while improving livestock health/nutrition and delivering multiple environmental co-benefits (Case study 1, Figure 1). Meanwhile, Malawi’s Small Scale Livestock and Livelihoods Programme hub integrates dual-purpose crops and ethnobotanical treatments, promoting animal health, reducing reliance on synthetic inputs, and substantially improving smallholder incomes (Case study 2, Figure 1). These practices also promote One Health by reducing inappropriate antibiotic use and supporting antimicrobial stewardship (via training, hygiene, and veterinary access). These are not isolated wins; they address intertwined issues like climate resilience, evident in India’s Silent Valley (Kerala Veterinary and Animal Science University), where heat-tolerant Vechur cattle and cooling systems mitigate heat stress amid rising temperatures. The GFP’s longevity, unlike other short-term funded research initiatives, is attributed to its delivery and multi-actor approach which rejects one-size-fits-all solutions, instead tailoring practices to cultural and ecological nuances, ensuring ruminant systems deliver public goods like carbon sinks—through well-managed grazing of perennial forages and integrated crop-livestock rotations that enhance soil organic carbon sequestration, nutrient cycling, and soil health—and nutritious food without compromising planetary health.

## Scaling through Spokes: From Research to Real-World Impact

The true power of the hub-and-spoke model lies in its scalability: hubs generate evidence, but spokes propel it into action across farms and regions. At Future Farm (Harper Adams University) in the UK, innovations like nutrient management from livestock waste are trialed and rapidly disseminated through the Sustainable Farm Network to thousands of commercial farms (spokes) leading to more rapid adoption and impact (Case study 3, Figure 1). In Kenya’s Kapiti ranch at the International Livestock Research Institute, genetically superior animals and grazing strategies reach pastoralist associations, enhancing herd resilience and informing policies in neighboring countries. Partnerships amplify this: collaborations with governments (e.g., USDA, UK’s DEFRA), industry (e.g., DairyNZ, Arla), and civil society (e.g., Farm Africa, CGIAR) facilitate workshops, internships, and train thousands globally (e.g., a UWA course alone reaching 42,000 learners). Economically, this yields dividends, like China’s CAU (China Agricultural University) boosting pastoralist incomes by 450 yuan per hectare via planned grazing. Socially, it empowers marginalized groups through improved market participation and decision-making opportunities (Case study 2, Figure 1). Environmentally, outcomes like SRUC’s (Scotland’s Rural College) genetic improvement programs in hill sheep, predicted to deliver 1–2% per annum cumulative reductions in methane emissions through selective breeding, demonstrate scalable climate action in extensive upland systems. In our opinion, this networked scaling transforms abstract research into livelihood lifelines, proving that sustainable ruminant production is not a luxury, it is achievable through inclusive collaboration (Rivero et al., 2021).

## Policy Implications and Future Horizons

The hub-and-spoke network’s ripple effects extend to policy, providing evidence that shapes frameworks like Kenya’s Livestock Master Plan or EU agrobiodiversity policies from Belgium’s Hooibeekhoeve. By aligning with FAO’s Livestock Sustainable Transformation vision, sustainable production, welfare, and One Health, it offers a roadmap for global transformation. GFP studies (Akpensuen et al., 2025) strongly support integrating climate-smart grassland policies into African livestock and land-use strategies. Africa’s vast grasslands, critical for rural livelihoods, face growing threats from climate variability and degradation. Climate-smart practices, such as diverse grass-legume-herb mixtures and adaptive pasture technologies, boost forage productivity, enhance soil health (via nitrogen fixation and carbon sequestration), increase resilience to extremes, stabilize livestock nutrition, and sustain ecosystem function. These approaches align with African policy goals for sustainable restoration, adaptive management, and investment in soil protection, food security, and climate resilience, while advancing net-zero agriculture. Scaling up cross-continental collaboration could accelerate progress toward the Sustainable Development Goals (SDG), i.e., SDG2—Zero Hunger, SDG13—Climate Action and SDG15—Life on Land. In our view, in an increasingly fragmented global landscape, collaborative networks such as the GFP’s hub-and-spoke approach offer a powerful mechanism for integration and progress. By providing robust evidence for sustainable ruminant production, they enable systems that meet human needs while preserving planetary health. Policymakers and stakeholders should prioritize investment in expanding such networks; the long-term viability of our food systems depends on it.

## Author Biographies

**Dr Jordana Rivero** is a researcher in Grazing Livestock Systems, inaugural Chair of the Global Farm Platform, and Principal Sustainability Consultant at Promar International (UK). Her work focuses on livestock grazing systems with an emphasis on sustainability, covering pasture and grazing management, livestock performance, animal behavior and welfare, forage quality, and breeding objectives for several livestock species. She holds an agricultural engineer degree from Uruguay, where she developed breeding objectives for dairy cattle under pasture-based systems, and a PhD in Agricultural Science from the Universidad Austral de Chile, gaining experience in grazing systems and NIRS applied to feeds and animal products. Her postdoctoral research examined the joint effects of agronomic management and perennial ryegrass cultivars on grazing dairy cows’ performance, nutrient use efficiency, and emissions.

**Dr Joshua Onyango** is a Lecturer in Veterinary Science, International Lead and Researcher at Harper and Keele Veterinary School (joint Veterinary school between Harper Adams University and Keele University), United Kingdom, and Vice-Chair of the Global Farm Platform. His research interests broadly focus on animal health and production, One Health, infectious and non-infectious diseases of veterinary and human importance. He studied animal health at Egerton University in Kenya, a BSc (Hons) at University of the West of England, an MSc in Animal Health and Production and a post graduate Certificate in Education both at University of Essex and a PhD in Veterinary Epidemiology from University of Northampton, United Kingdom where he looked at risk factors for uterine infections in English dairy herds and epidemiology of orf in English sheep flocks.

**Professor Michael Lee** is Deputy Vice Chancellor at Harper Adams University, England’s premier specialist agriculture and land use university. He is an expert in sustainable livestock systems, with a focus on defining their role in securing global food security while protecting environmental health (Livestock’s role in human and planetary health). He holds a first-class honors degree in Animal Science from the University of Wales, Aberystwyth, and a PhD in ruminant nutrition (protein and energy metabolism) from the University of Aberdeen. He also earned a Postgraduate Certificate in Teaching in Higher Education from Aberystwyth University. His research interests center on sustainable livestock production, ruminant nutrition, and the integration of livestock systems with environmental sustainability and food security objectives.
